# Steroidogenic Factor-1 (SF-1, NR5A1) and 46,XX Ovotesticular Disorders of Sex Development: One Factor, Many Phenotypes

**DOI:** 10.1159/000454806

**Published:** 2016-12-15

**Authors:** Kenneth McElreavey, John C. Achermann

**Affiliations:** ^a^Human Developmental Genetics, Institut Pasteur, Paris, France; ^b^Genetics and Genomic Medicine, UCL Great Ormond Street Institute of Child Health, London, UK

Gerard Conway, a colleague at the adult DSD clinic at UCLH, recently said: “If the phenotype doesn't make sense, think of SF-1.” This may not always be true, but steroidogenic factor-1 (SF-1/NR5A1)-related phenotypes have expanded greatly in recent years, and the report of Swartz et al. [1] in this issue of *Hormone Research in Paediatrics* adds to the emerging picture.

SF-1 (officially known as NR5A1, but hereafter called SF-1) was originally discovered by Keith Parker and Ken-Ichirou Morohashi in the early 1990s following the search for a master-regulator of steroidogenesis. SF-1 is an orphan nuclear receptor that controls many aspects of adrenal and reproductive development and function. Disruption of the gene encoding Sf-1 in the mouse results in adrenal underdevelopment and testicular dysgenesis. When 2 children with a similar phenotype were reported around the turn of the century, it seemed severe disruption of SF-1 would be an important differential diagnosis in paediatric endocrinology, but children with this condition would probably be extremely rare.

The past decade, however, has seen a massive expansion in both the number of reported children and families with clinically significant variations in SF-1 and the range of associated phenotypes [2]. Heterozygous loss-of-function variations in SF-1 have been described throughout the spectrum of 46,XY disorders of sex development (DSD), including testicular dysgenesis with or without müllerian structures, disordered androgen production, penoscrotal hypospadias, progressive androgenisation at puberty, and even male factor infertility. Although women can harbour SF-1 variants and pass them on in a sex-limited dominant fashion (that mimics X-linked inheritance), disruption of SF-1 is also associated with primary ovarian insufficiency and early menopause, although the penetrance of the phenotype is variable. Severe disruption of SF-1 can rarely be associated with primary adrenal insufficiency in 46,XX girls. However, to date, adrenal function has been preserved in the vast majority of individuals with heterozygous SF-1 variants, although long-term studies are needed to know whether this might deteriorate with age.

This year, the SF-1 story has taken a new twist, with several reports showing that alterations in SF-1 can be associated with 46,XX ovotesticular DSD (OTDSD) and 46,XX testicular DSD (TDSD) (“XX males”). Remarkably, all affected individuals had exactly the same change, a heterozygous p.Arg92Trp variant.

In the first published report, Bashamboo et al. [3] described 2 sisters with OTDSD, 2 men with TDSD, and a family with a 46,XX child raised male and a 46,XY child raised female. These individuals and families came from diverse genetic backgrounds: European, African, Hispanic, and South Asian. Shortly afterwards, Baetens et al.[4] reported 3 apparently unrelated individuals with OTDSD or TDSD from Belgium/the Netherlands, and Igarashi et al.[5] described 2 unrelated individuals with OTDSD/TDSD in Japan. Codon 92 is a critical amino acid in the “A-box” accessory DNA-binding region of SF-1. Laboratory studies suggest that the p.Arg92Trp variant alters repression of testis pathways, although data in robust biological systems are limited. Indeed, insertion of the specific p.Arg92Trp change into the mouse by CRISPR/Cas9 genome-editing does not produce a phenotype in the ovary [6]. Although many changes had arisen de novo, penetrance of the phenotype in humans is variable, with several 46,XX female carriers of the p.Arg92Trp change seemingly unaffected.

The report by Swartz et al. [1] in this edition of *Hormone Research in Paediatrics* adds to the intrigue of the situation. Using whole-exome sequencing of individuals with OTDSD, they have identified a heterozygous p.Arg92**Gln** variant (not p.Arg92Trp) in a child with 46,XX OTDSD of American-European ancestry. This variant was inherited from her unaffected father. Of note, this specific change has been reported previously in a homozygous state in association with 46,XY DSD and adrenal insufficiency as well as in a 46,XX girl with adrenal insufficiency alone [7, 8]. Both these children came from Northern Turkey, and heterozygous carriers so far have been reported to be unaffected [5, 7].

These studies are providing fascinating insight into the role of SF-1 in human endocrine development but are also raising many questions. Variations in codon 92 can clearly alter human ovary development resulting in partial or complete testis development. The report of 11 children and adults with OTDSD/TDSD in the past few months alone, the de novo nature of the changes in many cases, and the complete absence of these variants in control databases all make this association genetically robust. However, many questions remain about the exact biological mechanism (or mechanisms) involved and why the phenotypic penetrance is variable. The human gonad is exquisitely sensitive to gene dosage effects during critical windows of development, possibly much more so than the mouse gonad. Other genetic, epigenetic, or environmental modifiers or somatic changes may also influence the phenotype. Subtle imbalance of a sex-determining “switch” at a critical time in embryo development could have a big effect. Of course, this makes our task of providing appropriate information and counselling for young people and their families all the more difficult, but this is a challenge we must embrace. SF-1-related conditions are now an important part of paediatric endocrinology, and having multiple phenotypes associated with different variations in the same gene is a phenomenon that we are starting to observe for many genes, not just SF-1.

## Disclosure Statement

The authors have no conflicts of interest to declare. J.C.A. is a Wellcome Trust Senior Research Fellow in Clinical Science (grant No. 098513/Z/12/Z).
